# Independent and combined effects of lifestyle behaviors on adolescent health-related quality of life

**DOI:** 10.1590/0034-7167-2022-0780

**Published:** 2023-10-09

**Authors:** Dartagnan Pinto Guedes, Bruna Hatsue Santos Yamaji, Marizete Arenhart Zuppa

**Affiliations:** I Universidade Estadual do Norte do Paraná. Jacarezinho, Paraná, Brazil; II Universidade do Oeste de Santa Catarina. São Miguel do Oeste, Santa Catarina, Brazil

**Keywords:** Adolescent Health, Adolescent Behavior, Health Behavior, Health Promotion, Health Education., Salud del Adolescente, Conducta del Adolescente, Conducta Sanitaria, Promoción de la Salud, Educación en Salud., Saúde do Adolescente, Comportamento do Adolescente, Conduta de Saúde, Promoção da Saúde, Educação em Saúde.

## Abstract

**Objective:**

To investigate the independent and combined effects of lifestyle behaviors, including physical activity, sedentary behavior, sleep duration and food intake, in the health-related quality of life (HRQoL) of Brazilian adolescents.

**Methods:**

Cross-sectional school-based study, with the participation of 306 adolescents aged 14 to 18 years. A questionnaire was applied with structured questions to collect lifestyle behaviors data. Perception of the HRQoL was identified using the Kidscreen-27. The study used covariance analysis and linear regression models for statistical analysis.

**Results:**

Adolescents who reported ≤ 2 hours/day of screen-based sedentary behavior and sleep duration equivalent to 8-10 hours/night presented significantly higher HRQoL. Adolescents who reported joint adherence ≥ 3 healthy lifestyle behaviors demonstrated approximately two [OR=2.12] to three times [OR=3.04] more chance of presenting higher perceptions of HRQoL.

**Conclusion:**

Although healthy lifestyle behaviors had a positive independent effect on HRQoL, joint adherence to healthy behaviors enhances the cumulative effect.

## RESUMO

**Objetivo:**

Investigar os efeitos individual e combinado de condutas do estilo de vida, incluindo atividade física, comportamento sedentário, sono e consumo alimentar, na qualidade de vida relacionada à saúde (QVRS) de adolescentes brasileiros.

**Métodos:**

Estudo observacional de base escolar, com participação de 306 adolescentes de 14 a 18 anos. Foi aplicado questionário com questões estruturadas para levantar dados de condutas do estilo de vida. Percepção da QVRS foi identificada por meio do Kidscreen-27. Análise de covariância e modelos de regressão linear foram usados para análise estatística.

**Resultados:**

Adolescentes que apontaram ≤ 2 horas/dia de comportamento sedentário baseado em tela e duração de sono entre 8-10 horas/noite apresentaram QVRS significativamente mais elevada. Adolescentes que relataram adesão conjunta ≥ 3 condutas saudáveis do estilo de vida demonstraram aproximadamente de duas [OR=2,12] a três vezes [OR=3,04] mais chance de apresentarem percepções mais elevadas de QVRS.

**Conclusão:**

Embora condutas saudáveis do estilo de vida apresentaram efeito individual positivo sobre a QVRS, adesão conjunta de condutas saudáveis potencializaram o efeito cumulativo.

## RESUMEN

**Objetivo:**

Investigar los efectos individuales y combinados de conductas del estilo de vida, incluyendo actividad física, conducta sedentaria, sueño y ingestión de alimentos, en la calidad de vida relacionada a la salud (CVRS) de adolescentes brasileños.

**Métodos:**

Estudio observacional de base escolar, con participación de 306 adolescentes de 14 a 18 años. Fue aplicada encuesta con cuestiones estructuradas para recoger datos de conductas del estilo de vida. Percepción de la CVRS fue identificada por medio del Kidscreen-27. Análisis de covarianza y modelos de regresión linear fueron usados para análisis estadístico.

**Resultados:**

Adolescentes que apuntaron ≤ 2 horas/día de conducta sedentaria basado en pantalla y duración de sueño entre 8-10 horas/noche tuvieron CVRS significativamente más elevada. Adolescentes que relataron adherencia conjunta ≥ 3 conductas saludables del estilo de vida demostraron aproximadamente de dos [OR=2,12] a tres veces [OR=3,04] más chance de presentar percepciones más elevadas de CVRS.

**Conclusión:**

Aunque conductas saludables del estilo de vida resulten en efecto individual positivo sobre la CVRS, adherencia conjunta de conductas saludables potencializa el efecto acumulativo.

## INTRODUCTION

Although there may be differences in its conceptual structure, there is agreement that the quality of life, more specifically the health-related quality of life (HRQoL), is considered a multidimensional construct, which includes self-perception of well-being in the physical, emotional, psychological and social domains influenced by the individual’s experiences, expectations, and convictions^(1)^. In this context, in the case of youths, the dimensions equivalent to HRQoL are especially appropriate to monitor the health status, considering the lower probability of identifying harm and diagnosing chronic diseases in this population group^(2)^.

Data on the HRQoL of the young population is also a relevant alternative for monitoring specific interventions and public health programs^(3)^. In primary health care actions, youths’ perception of HRQoL can help to identify subgroups with higher risk for well-being^(4)^. In addition, the follow-up of HRQoL in childhood and adolescence is noteworthy due to its future repercussions on the quality of life of adults^(5)^. Therefore, dimensions equivalent to HRQoL should receive special attention in health care from young ages.

Findings available in the literature seek to show that the HRQoL of youths is closely related to lifestyle behaviors. Appropriate practice of physical activity^(6)^, longer sleep duration^(7)^, and healthy diet^(8)^ were positively associated with HRQoL, while screen time was inversely associated with HRQoL^(9)^. However, although lifestyle behaviors analyzed in isolation in these studies have shown close identification with HRQoL, youths tend to adhere simultaneously to a conglomerate of behaviors^(10)^, which can modify the individual effect of each conduct. In fact, previous studies have suggested that simultaneous adherence to multiple lifestyle behaviors can enhance the personal health impact by interacting synergistically with each other^(11)^.

## OBJECTIVE

To identify the independent and combined effects of a set of lifestyle behaviors, including physical activity, sedentary behavior, sleep, and food intake, on the HRQoL of a sample of Brazilian adolescents.

## METHODS

### Ethical aspects

The Research Ethics Committee of the Universidade do Oeste de Santa Catarina (Platform Brazil - No. 3.412.665/2019) approved the study intervention protocols, according to ethical aspects for research with human beings, according to the Resolution of the Brazilian National Health Council (Conselho Nacional de Saúde).

### Design, period and place of study

This is an excerpt from the Health Promoting School Project, designed and implemented by the Federal Institute of Santa Catarina, São Miguel do Oeste Campus. The study chose adolescents enrolled only in this school unit due to the project’s longitudinal characteristics (experimentation of health education programs) and their representativeness in the universe of high school students in the western region of the state of Santa Catarina, Brazil. The study used the STROBE instrument to guide the study methodology. Findings of other variables addressed in the Project were disseminated in previous publications^(12)^.

### Population or sample; criteria of inclusion and exclusion

The sample consisted of schoolchildren of both sexes, aged 14 to 18 years, who were attending high school. The participation of the students in the study occurred due to their desire to participate in the experiment and with the parents’ or guardians’ authorization. To this end, all schoolchildren enrolled in the 2019 school year, together with their parents or guardians, were contacted and informed of the nature and objectives of the project, in addition to the principle of secrecy, not influencing school performance, and invited to participate in data collection. Refusal to participate in the study or not attending the invitation after three contact attempts on different days and times were considered sample losses.

The criteria adopted for the exclusion of a student from the study were: (a) have some health problem that could temporarily or definitively prevent participation in the study; (b) use of some type of medicine that could induce changes in study variables; (c) being on some type of specific diet; (d) pregnancy; and (e) inadequate filling of items in the measuring instrument (more than one answer for the same item or unanswered item). Thus, of the 418 students attending school, the definitive sample consisted of 306 adolescents (179 girls and 127 boys). The rights of all participants were protected by the Free and Informed Consent Form signed by the students and their guardians.

### Measuring instrument

For the data survey, the study applied a questionnaire consisting of three sections: demographic data, HRQoL, and lifestyle behaviors. Concerning demographic data, in addition to sex and age, it collected information on the year of study, parents’ schooling level, and family economic class based on housing conditions, household utensils, cars, and number of domestic employees, according to Brazil’s classification criteria, recommended by the Brazilian Association of Research Companies guidelines^(13)^.

The researchers translated, adapted and validated the Kidscreen-27 to examine the HRQoL domains for use in the young Brazilian population^(14)^. Participants who presented an overall HRQoL score above the sex-specific normative value, originally proposed by the creators of Kidscreen questionnaires (girls = 49; boys = 51), were classified as having a high HRQoL^(15)^.

Information equivalent to lifestyle behaviors was obtained through items equivalent to physical activity, sedentary behavior, sleep, and food intake. The physical activity was identified by the formulation of the question: “In the last seven days, how often have you performed moderate to vigorous physical activity for at least 60 minutes? The answer options for the question were “None” to “7 days”. Following international public health guidelines, those adolescents who responded to physical activity frequency from moderate to vigorous intensity for at least 60 minutes in 7 days/week were considered sufficiently active^(16)^.

Sedentary behavior was treated by exposure to recreational screen time through the question: In a typical or usual week, how many hours do you watch TV and/or use a computer, tablet, or smartphone for activities that are not related to any kind of work or school task? A predefined time scale was provided for response, in which the respondents indicated their options between six categories, ranging from “none” to “≥ 5 hours/day”. The question considered the use of screen devices equivalents to weekdays and weekends separately. Weighted average involving the data of days of week and weekends was used to identify the screen time per day. In this case, according to scientific societies linked to the health of youths, adolescents who reported a mean screen time ≤ 2 hours/day were considered to have the least sedentary behavior^(17)^.

The study also collected data equivalent to sleep duration, considering days of week and weekends, regarding a typical or usual week, using the following questions: On weekdays and weekends: (a) what time do you usually sleep? (b) and what time do you wake up? A weighted average of the weekday and weekend data was used to identify sleep duration per night. For the purpose of analysis, sufficient sleep was attributed to those adolescents who reported a duration of 8-10 hours/night^(18)^.

Regarding food intake, the participants positioned how often they consumed fruit/vegetables through the question: “In the last seven days, how often have you eaten fruits and/or vegetables (do not consider fruit juices)?” The answer options ranged from “none” to “seven days*.”* Appropriate fruit/vegetables intake was attributed to adolescents who reported responses equivalent to seven days/week^(19)^.

Subsequently, the global healthy lifestyle behaviors index was generated by combining the four health behaviors considered in the study (physical activity, sedentary behavior, sleep, and food intake). Thus, adolescents received a score for each reported healthy behavior. Hence, the score of the global healthy lifestyle behaviors index ranged from zero to 4, where zero represents the absence of any of the health behaviors, and the other scores represent the number of behaviors present simultaneously, where higher scores indicate a healthier lifestyle.

### Data collection

Data were collected between September and November 2019 by a team of researchers who knew the instrument and were trained in its procedures. The questionnaire was answered in a single moment, individually by each of the participants, and at the place and time of the class. The participants received the questionnaire with instructions and recommendations for self-completion, and no time limit was established for completion. The average time to complete the questionnaire was 40 minutes. The questionnaire reliability was analyzed by reapplying it to 10% of the participants seven days later. All the items presented a Cohen concordance index ≥ 0.80.

### Statistical treatment

The IBM® SPSS® Statistics for Windows Package, version 28 (IBM Corporate, Armonk, New York, USA) conducted the data analysis. Regarding the scores for the five domains and the overall HRQoL, the Kolmogorov-Smirnov test initially analyzed the frequency distribution. Considering that they showed a normal frequency distribution, the study used mean and standard deviation calculations. Subsequently, to establish comparisons between both sex, the Student’s t-test was used for non-paired data. Regarding lifestyle behaviors, the analysis identified specific proportions, and respective confidence intervals (CI_95%_) stratified according to sex. Statistical differences among the strata under investigation were analyzed using contingency tables and chi-square non-parametric test (χ^2^).

As preliminary analyzes did not identify significant interactions of sex with lifestyle behaviors concerning the HRQoL domains, in the sequence, researchers performed statistical procedures involving the data set of both sexes. Comparisons among the scores equivalent to the overall HRQoL categorized in the strata of each lifestyle behavior were made using analysis of covariance (ANCOVA), adjusted by sex, age, year of study, parents’ schooling, and family economic class. In addition, ANCOVA accompanied by Bonferroni’s post hoc test, was used to identify specific differences in overall HRQoL scores among the five healthy behavior indices of lifestyle. The partial eta-squared (η^2^p) was calculated to analyze the effect size^(20)^.

Logistic regression analysis procedures, adjusted for sex, age, year of study, parents’ schooling, and family economic class, were used to estimate the probability of adolescent schoolchildren presenting high HRQoL according to healthy lifestyle behavior indices. Statistical significances were pre-established in p < 0.05.

## RESULTS

The study participants had an average age equivalent to 16.34 ± 1.21 years. Table 1 provides statistical information equivalent to the domains of HRQoL and lifestyle behaviors separately by sex. The boys attributed significantly higher scores in the domains of HRQoL related to physical well-being (p < 0.001), psychological well-being (p = 0.023), and autonomy/parent relations (p = 0.011). However, average scores identified in the peers/social support and school environment domains were similar in both sexes. Concerning the overall index of HRQoL, boys also scored more than girls (p = 0.048). Regarding the exposure to lifestyle behaviors, both sexes reported similar behavior of screen time-based sedentary behavior and sleep duration. However, a significantly higher proportion of boys were more physically active (p < 0.001), while a higher proportion of girls showed more favorable fruit/vegetables intake (p = 0.007).

**Table 1:** Descriptive characteristics of the domains of health-related quality of life and lifestyle behaviors according to adolescents’ sex participating in the study

	Both sexes	Girls	Boys	*p*
**Health-related quality of life (mean ± standard deviation)^1^**				
Physical well-being	57.10 ± 13.51	54.34 ± 13.57	60.98 ± 13.64	< 0.001
Psychological well-being	61.07 ± 17.05	59.43 ± 16.78	63.39 ± 19.39	0.023
Autonomy/parent relations	65.28 ± 18.86	63.65 ± 21.51	67.57 ± 17.32	0.011
Peers/social support	71.60 ± 16.32	71.89 ± 16.49	71.20 ± 16.83	ns
School environment	57.61 ± 15.59	58.50 ± 15.68	56.37 ± 15.87	ns
Overall HRQoL	62.53 ± 16.37	61.56 ± 16.21	63.90 ± 16.61	0.048
**Lifestyle Behaviors (% _[CI95%]_)^2^**				
Physical activity	21.2	15.6	28.3	< 0.001
7 days/week	[19.1 - 23.7]	[14.4 - 17.2]	[25.1 - 31.9]	
Screen time-based sedentary behavior	22.2	24.0	19.7	ns
≤ 2 hours/day	[20.0 - 24.9]	[21.6 - 26.9]	[17.7 - 21.8]	
Sleep duration	34.3	33.0	36.2	ns
8-10 hours/night	[30.5 - 38.8]	[29.4 - 37.2]	[31.5 - 40.9]	
Fruit/vegetables consumption	29.7	33.5	24.4	0.007
7 days/week	[26.7 - 33.0]	[29.8 - 37.5]	[22.0 - 27.3]	

1 Comparison between both sexes by Student’s t test.

2 Comparison between both sexes by chi-square test.

The ANCOVA results comparing the scores equivalent to the overall HRQoL according to strata of the individual lifestyle behaviors are available in Table 2. Adolescents who reported ≤ 2 hours/day of screen time-based sedentary behavior (F = 5.496; p = 0.016) and sleep duration between 8-10 hours/night (F = 6.542; p = 0.009) presented scores equivalent to the overall HRQoL significantly higher. However, in the strata that gathered the adolescents who reported being more physically active or consuming more frequently fruits/vegetables, the analysis did not identify significant effects on scores equivalent to the overall HRQoL.

**Table 2:** Individual effects of lifestyle behaviors on overall index of health-related quality of life (Overall HRQoL) in adolescents

Lifestyle Behaviors	Overall HRQoL	F Test
Physical Activity		F = 3.075; p = 0.061
≤ 6 days/week	60.97 ± 15.75	
7 days/week	64.13 ± 16.99	
Screen time-based sedentary behavior		F = 5.496; p = 0.016
> 2 hours/day	59.78 ± 15.57	
≤ 2 hours/day	65.28 ± 17.09	
Sleep duration		F = 6.542; p = 0.009
< 8 hours/night	59.15 ± 15.49	
8-10 hours/night	65.91 ± 17.25	
Fruit/vegetables intake		F = 1.213; p = 0.185
≤ 6 days/week	61.90 ± 16.20	
7 days/week	63.16 ± 16.54	

Note: Covariance analysis adjusted by sex, age, year of study, parents’ schooling, and family economic class.

**Figure 1:**
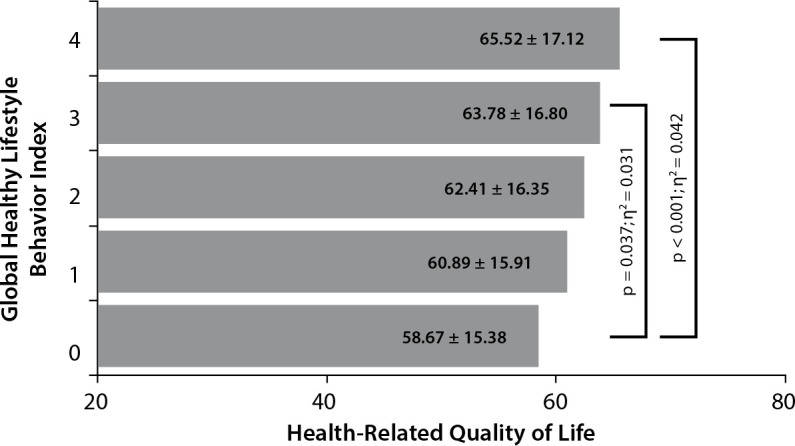
Health-related quality of life according to the global healthy lifestyle behavior

Mean scores equivalent to HRQoL according to the global healthy lifestyle behavior index are presented in Figure 1. The analyzes showed significant differences in HRQoL scores among the strata of the global healthy lifestyle behavior index (F = 8.472; p < 0.001; ƞ^2^p = 0.09) after adjustment for sex, age, year of study, parents’ schooling, and family economic class. Specifically, adolescents with a global healthy lifestyle behavior index of 3 and 4 presented higher HRQoL scores than their peers with a global healthy lifestyle behavior index equivalent to 0 (65.52 ± 17.12 versus 58.67 ± 15.38; p < 0.001; and 63.78 ± 16.80 versus 58.67 ± 15.38; p = 0.037; respectively).

Estimates of the probability of adolescents presenting high HRQoL according to the global healthy lifestyle behavior index are presented in Table 3. The odds ratio values indicated that adolescents who reported joint adherence to three or four healthy behaviors (indices 3 and 4) were more likely to present high HRQoL than their peers who reported not adhering to any of the four healthy lifestyle behaviors (index 0). Adolescents stratified in indices 3 and 4 of healthy lifestyle behaviors showed approximately two [OR = 2.12; CI_95%_ 1.27 - 4.79] to three times [OR = 3.04; CI_95%_ 1.93 - 5.62] more chances of presenting a high perception of HRQoL compared to their peers with index 0.

**Table 3:** Odds ratio (OR) and 95% confidence interval (CI_95%_) for the association between high health-related quality of life and global healthy lifestyle behavior index in adolescents

Global healthy lifestyle behavior index		High health-related quality of life	
	n	OR (CI95%)	*p*
0	23	Reference	
1	62	1.58 (0.62 - 3.34)	0.106
2	87	1.84 (0.96 - 4.08)	0.078
3	74	2.12 (1.27 - 4.76)	0.024
4	60	3.04 (1.93 - 5.62)	< 0.001

Note: Adjusted values for sex, age, year of study, parents’ schooling and family economic class.

## DISCUSSION

It was the first study that investigated the independent and combined effects of four classic lifestyle behaviors on the HRQoL of school-age adolescents in Brazil. The main results revealed that the combination of lifestyle behaviors, including higher frequency of moderate to vigorous physical activity for at least 60 minutes/day, average screen time ≤ 2 hours/day, sleep duration between 8-10 hours/night, and daily fruit/vegetables intake, positively influenced the HRQoL of the adolescents who participated in the study. Additionally, the greater the number of healthy behaviors reported by the adolescents, the greater the probability of presenting higher HRQoL. These findings seek to contribute to the scarce scientific literature on the subject, suggesting that the combination of multiple healthy lifestyle behaviors tends to have a more prominent impact on HRQoL than their respective independent effects.

When treating each of the lifestyle behaviors separately, the physical activity from moderate to vigorous intensity did not show an independent association with HRQoL. However, previous studies have pointed out significant associations between habitual physical activity and HRQoL^(6)^. In addition, adolescents who are more physically active tend to present a lower risk for depressive symptoms^(21)^, better cardiometabolic status^(22)^, sleep restoration and psychological condition^(23)^, which possibly may influence HRQoL. Thus, although no individual association was found between moderate and vigorous physical activity and HRQoL, it is likely that, combined with other healthy lifestyle behaviors investigated, it may also impact HRQoL^(24)^.

In line with data available in the literature^(9)^, the study results showed that adolescents who reported ≤ 2 hours/day of screen time presented a higher perception of HRQoL. These results may be related to passivity and the solitary context of screen activities, which may restrict or replace social interaction with peers and do not imply situations that require problem resolution, and cognitive or physical challenges^(25)^. This behavior may influence the degree of satisfaction with life, psychological well-being, and physical health status, which, in turn, negatively influences the HRQoL perception^(26)^.

On the other hand, the association observed between longer sleep (8-10 hours/night) and higher self-reported HRQoL supports results from previous studies and confirms a positive individual effect of sleep duration in adolescents’ HRQoL^(7)^. These findings can be partially explained by the direct consequence of daytime sleepiness due to insufficient sleep^(27)^. Daytime sleepiness can induce a reduction in the state of alert and compromise functional capacity, including fatigue, mood alterations, performance reduction in daily tasks, memory impairment, and difficulty in dealing with adversities^(28)^. Thus, the daytime losses resulting from reduced sleep duration influence cognitive, physical, and emotional performance throughout the day, which may, in turn, impact on adolescents’ HRQoL^(29)^. As for food consumption, the results of the present study agree with the facts available in previous surveys^(30)^, which also did not show significant differences in the HRQoL of adolescents who reported daily fruit/vegetables intake and adolescents who did not informed this dietary pattern.

The combined effect analysis revealed that adolescents with a global healthy lifestyle behavior index ≥3, compared to those with a global index equivalent to 0, presented higher mean HRQoL scores with a moderate effect, as denoted by the effect size obtained. In addition, a cumulative effect of healthy lifestyle behaviors was found on HRQoL. Although the results found may suggest that not all healthy lifestyle behaviors included in the current study demonstrate the same impact on adolescents’ HRQoL, the analyzes combined effects revealed that the greater the adherence to healthy behaviors, the higher the HRQoL.

Few studies have analyzed the combined effects of multiple lifestyle behaviors on the HRQoL of adolescents to date^(31-32)^. The available studies included in their design lifestyle behaviors similar to those used in the present study and identified that adolescents with healthy lifestyle behaviors self-reported significantly higher. Therefore, this study’s results corroborate these findings, confirming that adherence to multiple healthy lifestyle behaviors is closely associated with higher HRQoL levels. Nonetheless, the present study added an unprecedented finding by revealing an increased probability of self-reporting due to a greater perception of HRQoL as adherence to healthy lifestyle behaviors also increases. In principle, these findings can be explained by the interaction of the positive effects that some healthy lifestyle behaviors present, which enhances the cumulative effect on adolescents’ HRQoL.

### Study limitations

Among the limitations, although the study implemented strict quality control to minimize possible inaccuracies, the data on the multiple lifestyle behaviors and the HRQoL were collected through self-report, thus allowing for possible tendentious biased towards the desirable. Furthermore, the cross-sectional nature of the data may limit the inferences of the long-term effects of lifestyle behaviors in HRQoL because the outcome and independent variables were identified at the same time, increasing the risk of inverse causality bias. Moreover, although the study used adjustments for some demographic data of the participants, residual confounding caused by other unidentified and unmeasured data may, in some way, enhance eventual inaccuracy of the findings. Another relevant aspect to be mentioned is the fact that the choices of lifestyle behavior during adolescence are strongly determined by the family environment and the sociability with the peers^(33)^, and these variables were not considered in the analyzes, which may also eventually have contaminated the findings.

### Contributions to the fields of Nursing and Health

The present study tried to combine four lifestyle behaviors in a global healthy lifestyle behavior index to provide a better understanding of the effects of these health behaviors on adolescents’ HRQoL. Considering the cumulative effect of adopting multiple healthy behaviors, it would be advisable for public health strategies to focus on promoting multi-behavioral health policies. In this context, this is especially relevant in adolescence, considering that it is a stage in the youths’ life especially favorable to the adherence to healthy lifestyle behaviors, with critical repercussions for later ages, thus influencing the current and future health of the adult^(10)^.

## CONCLUSION

The study results showed that sleep duration between 8-10 hours/night and screen time-based sedentary behavior ≤ 2 hours/day presented positive independent effects on the adolescents’ HRQoL. However, the combined effects of healthy lifestyle behaviors associated with physical activity, screen time-based sedentary behavior, sleep duration, and food intake showed a more substantial influence on HRQoL. Therefore, educational and public health interventions focused on promoting HRQoL among adolescents should focus on multiple actions related to healthy lifestyle behaviors. The importance of the study findings is highlighted due to the fundamental role of maintaining a high HRQoL since the earliest ages and throughout life.
